# Historical records of plant-insect interactions in subarctic Finland

**DOI:** 10.1186/s13104-022-06213-x

**Published:** 2022-10-08

**Authors:** Leana Zoller, Tiffany M. Knight

**Affiliations:** 1 Institute of Biology, Martin Luther University Halle-Wittenberg, Am Kirchtor 1, 06108 Halle (Saale), Germany; 2 German Centre for Integrative Biodiversity Research (iDiv) Halle-Jena-Leipzig, Puschstraße 4, 10, 04103 Leipzig, Germany; 3 Department of Community Ecology, Helmholtz Centre for Environmental Research- UFZ, 12 Theodor- Lieser-Straße 4, 06120 Halle (Saale), Germany

**Keywords:** Pollination, Climate change, Decade, Long-term, Plant-pollinator interactions, Interaction network

## Abstract

**Objectives:**

Historical ecological records document the diversity and composition of communities decades or centuries ago. They can provide a valuable benchmark for comparisons with modern communities. Historical datasets on plant-animal interactions allow for modern comparisons that examine the stability of species and interaction networks over long periods of time and in response to anthropogenic change. Here we present a curated dataset of interactions between plants and insects in subarctic Finland, generated from digitizing a historical document from the late 19th century and updating the taxonomy using currently accepted nomenclature.

**Data description:**

The resulting dataset contains 654 records of plant-insect interactions observed during the years 1895–1900, and includes 498 unique interactions between 86 plant species and 173 insect taxa. Syrphidae, Apidae and Muscidae were the insect families involved in most interactions, and interactions were most observed with the plant species *Angelica archangelica*, *Salix caprea*, and *Chaerophyllum prescottii*. Interaction data are available as csv-file and provide a valuable resource on plant-insect interactions over 120 years ago in a high latitude ecosystem that is undergoing rapid climate change.

## Objective

The rapid degradation of natural ecosystems in the Anthropocene [1, 2] highlights the increasing need for conservation actions that preserve life-sustaining ecosystem functions and services [3]. Pollination is a vital ecosystem service as most angiosperm plants, including many crops, rely on animal pollination for sexual reproduction [4, 5]. There have been recent observations of declines of pollinators and the plants they are associated with [6], driven by intensive agriculture, pesticides, the spread of invasive species and pathogens, and climate change [7]. It may take decades or centuries for the full effects of these drivers on plant-pollinator interactions to be realized, and short-term studies may therefore underestimate their effects. Currently, our knowledge on temporal and spatial changes in plant-pollinator interactions is limited, as the vast majority of studies documenting plant-pollinator interactions encompass only one or a few years of the present [8] and come from North America and Western Europe [9].

One way to bridge this knowledge gap is through the use of historical records on plant-pollinator interactions. Historical datasets documenting these interactions (i.e. insects coming into contact with the reproductive organs of flowers) are rare, but provide unique opportunities to examine long-term changes in pollinator communities and the structure of plant-pollinator networks, enabling many modern research questions in pollination ecology. Data from arctic and subarctic regions are particularly valuable, because these regions are experiencing more rapid climate change compared to the global average [10] and understudied flies are the most important pollinators there [11, 12]. Here, we present a digitized and curated dataset on plant-insect interactions in subarctic Finland derived from a historical document.

## Data description

During six years, between May and August of the years 1895–1900, Frans Silén documented interactions between plants and insects in Kittilä, Finland and published these observations in the naturalist journal Meddelanden af Societas pro Fauna et Flora Fennica [13]. Kittilä is located ~ 120 km north of the Arctic Circle in a boreal biome (67.66 Lat.; 24.89 Long.). Silén’s original publication consists of a list of 654 records of 86 plant species visited by a total of 173 insect taxa, resulting in 498 unique interactions.

In a first step, all of Silén’s original records were manually digitized. Each unique plant-insect interaction per site and date was entered as a new row of data (hereafter referred to as ‘record’). Full verbatim taxonomic species names of plants and pollinators (as originally stated in the historical document), verbatim locality and date (year, month and day) were included. Additional information on insect sex (i.e. m/f), insect behaviour (e.g. nectar sucking) and categorical abundance (e.g. “scarce”, “many”) was available for many records. Some records in the historic document contained additional comments or field notes which were also included in the dataset. In a second step, verbatim taxonomic plant and insect names were updated to currently accepted names and added to the interaction dataset. Each unique verbatim taxonomic name was cross-checked with the GBIF Backbone Taxonomy and/or Finnish species checklists and, if necessary, the taxonomic name was updated to the currently accepted name (according to the GBIF Backbone taxonomy). Additionally, we extracted information on order, family, and genus of each taxon. When verbatim taxonomic names could not be resolved to a valid taxon using the GBIF Backbone Taxonomy and checklists, we manually researched taxonomic revisions of the verbatim taxa in other databases, publications or checklists. When the verbatim species names could not be resolved to any currently valid species, the next finest available resolution (genus, family or order), was recorded. Further, we verified if the derived species have previously been reported from Finland using the online portal (laji.fi) of the Finnish Biodiversity Information Facility (FinBIF). Verbatim taxonomic names with corresponding updated names, sources for the new names, and information of occurrence in Finland as well as the GBIF identifiers of each taxon are provided for plants and insects in two supplementary data files [14] (Table 1).

After cross-checking taxonomic names, 153 insect taxa were resolved to species level (94.34% of records), 13 to genus (2.60% of records), six to family (2.14% of records) and one to order level (0.92% of records). All plant species could be resolved to species level. The recorded insect species belong to four orders (Diptera, Hymenoptera, Lepidoptera and Coleoptera) and include 88 genera in 30 families. The most frequently recorded insect families were Syrphidae, Apidae and Muscidae and the most frequently recorded genera were *Bombus, Platycheirus* and *Thricops.* Salicacea, Apiaceae and Asteracea were the most frequently recorded plant families, and in particular the plant species *Angelica archangelica, Salix caprea*, and *Chaerophyllum prescottii.*

**Table 1:** Overview of data files/data sets

Label	Name of data file/data set	File types (file extension)	Data repository and identifier (DOI or accession number)
Data set 1	Historical records of plant-insect interactions in subarctic Finland		figshare: 10.6084/m9.figshare.c.5828663.v4 [15]
Data file 1	InteractionData_Silen.csv	csv-file	figshare: 10.6084/m9.figshare.19130474.v4 [16]
Data file 2	Supplementary data files for: Historical records of plant-insect interactions in subarctic Finland	csv-files	figshare: 10.6084/m9.figshare.19130501.v2 [14]

## Limitations

As important and valuable as historical data are, working with them often presents significant challenges and limitations. A thorough examination of the potential limitations, and methods to minimize them, is therefore required. The main limitations of the dataset presented here are that methodology, sampling effort and sampling conditions (e.g. time of day, weather) are incompletely described in the historical source. For example, it is not known whether the observation of flower visitors was conducted using standardized methods or if it was done opportunistically. It is also unclear what the sampling effort was for each plant species and whether it was comparable for all plant species. However, potential biases introduced by these limitations can be minimized by using appropriate resampling methods and statistical measures. For example, using a combination different resampling approaches (i.e. individual-based and plot-based sampling) can minimize methodological biases, and standardizing data by number of individuals or sampling completeness can minimize biases due to differences in sampling effort.

## Data Availability

The data described in this Data note can be freely and openly accessed on figshare under 10.6084/m9.figshare.c.5828663.v4 [14–16]. Please see Table 1 for details and links to the data.
